# Breaking the glass door in academia? Looking at the role of scientific fields and contextual factors in moderating the gender gap in recruitment: evidence from Italy

**DOI:** 10.1007/s10734-025-01466-4

**Published:** 2025-07-15

**Authors:** Camilla Gaiaschi

**Affiliations:** University of Salento, Lecce, Italy

**Keywords:** Academic careers, Glass door, Gender discrimination, STEM, Early career researchers, Gender and science

## Abstract

Women’s presence in academia has sharply increased in recent years, but gender inequalities in career progression persist. Italy makes no exception to this trend as different studies on the promotion gap to associate and to full professors have suggested. However, no measure of the adjusted gender differential in the previous steps of the career, that is in the transition from the post-doc to the assistant professor position, has been provided so far. This paper aims to fulfill this gap by means of an original, longitudinal, and multi-source dataset on the Italian academic population which has allowed to measure the female disadvantage in recruitment by controlling for a large set of confounding characteristics including individual productivity and, most especially, contextual factors, which have been under-explored so far. Results suggest that women face a small adjusted penalty, of around 3–4%, in academic recruitment. However, when disentangling the analyses by scientific field, strong differences emerge, with the gap reaching a maximum of − 10% in physics while being non-significant in many SSH. Within the STEMM, the life sciences, driven by medicine and biology, appear more gender unequal than many hard sciences. Moreover, a growing number of female full professors in the sub-field and working in a department with good financial resources represent two factors that have a moderate role in reducing the gap. All in all, this work sheds light on the importance of organizational and institutional factors in explaining the gender gap thus calling for structural interventions to make universities more inclusive towards women.

**Supplementary Information:**

The online version contains supplementary material available at 10.1007/s10734-025-01466-4.

## Introduction

Women’s presence in academia has sharply increased in recent years (American Council on Education, 2017; European Commission, 2024), but gender inequalities, both in recruitment and career advancement, persist. In Europe, the last She Figures report published by the European Commission confirms that parity has approximately been reached at the PhD level, with women representing 47.6% of doctorate graduates in 2021, a percentage that has remained stable in the last ten years. However, their presence progressively decreases all along the career ladder, a phenomenon known as “leaky pipeline” (Alper, 1993), with women representing 46.8% of grade C, 42% of grade B, and 29.7% of grade A academic staff (European Commission, 2024).^1^ Italy makes no exception to these figures, with 48.9% of female PhD holders, 45.6% of grade C, 42.3% of grade B, and 27% of grade A academic staff in the same year according to the same report. When considering the STEM fields, the gender unbalance is apparent since the very early steps of the career ladder, with women being only 37% of PhD graduates, 35% of grade C, 29.5% of grade B, and 20.3% of grade A academic staff (European Commission, 2024).

If descriptive statistics represent a good instrument to understand if and where, across positions and scientific fields, gender inequalities persist, they are not enough to identify if mechanisms of discrimination, which occur when equivalent men and women are differently treated in terms of recruitment and/or promotion, continue to exist. For this purpose, multivariate regression-based studies using observational data or, even more so, experimental research designs, are needed. On this point, four recent observational studies conducted on the Italian academic population indicate that, net of unobserved characteristics, the likelihood for women to be promoted remains lower than that of men with equal attributes, including scientific productivity, both when it comes to becoming associate (De Paola et al., 2018; Filandri & Pasqua, 2021) and full professor (De Paola et al., 2018; Filandri & Pasqua, 2021; Marini & Meschitti, 2018; Meschitti & Marini, 2023). As such, these researches provide insights on, respectively, the so-called mid-level bottleneck (Yap & Konrad, 2009) and glass ceiling (Federal Glass Ceiling Commission, 1995) phenomena, which refer to the presence of gender obstacles to promotion at the intermediate (the former) and final (the latter) steps of the career ladder. Other studies have focused on survey-data on PhD holders and their work outcomes, outside and inside academia, thus looking at the female disadvantage during early career stages (Bozzon et al., 2017; Carriero & Naldini, 2022; Carriero et al., 2024). However, none of them specifically focuses on the transition from the post-doc to the assistant professor position, also called the “glass door” phenomenon (Picardi, 2019). Up to now, contributions analyzing this specific step of the Italian academic career ladder rely on descriptive accounts only (Gaiaschi & Musumeci, 2020; Picardi, 2019). No measure of the adjusted gender gap in the transition from post-doc to assistant professor has been provided so far with respect to the Italian context. Against this background, this paper aims to fulfill this gap by focusing on two main objectives: first, to measure the net female disadvantage in the transition from post-doc to assistant professor, second to look at its variation across a set of contextual factors, more precisely across (1) scientific fields, (2) levels of feminization of full professorship, and (3) department’s performance.

To do so, an agreement with the Italian Ministry of University and Research (MUR) within the frame of the WIRED project was made in order to access administrative panel micro-data on the Italian academic population, including post-docs. Parallel to administrative data, web-scrapped information on organizational performance was added as well. The final dataset includes extensive information tracked across time, both in term of individual (socio-demographic and work-related) characteristics and organizational factors, on post-docs and assistant professors working in Italy from 2010 to 2020. This has allowed not only to measure the gender gap in the probability to become assistant professor by controlling for a large set of confounding characteristics—thus providing, for the first time, an adjusted measure of post-docs’ disadvantage in accessing stable positions—but also to focus on the contribution of contextual factors which have been under-explored so far.

## Literature

Women’s disadvantage in academic career progression is well documented by a wide international literature. With few exceptions (see: Webber and González Canché, 2018; Carlsson et al., 2021), most of these studies indicate that women experience an adjusted penalty in the likelihood to become *full professor* (e.g. Durodoye et al., 2020), *associate professor* (e.g. Box-Steffensmeier et al., 2015; Weisshaar, 2017), and *assistant professor* (e.g. Ginther & Kahn, 2009). More recent contributions have focused on increasing academic precarity suggesting that women experience longer periods of job instability (Box-Steffensmeier et al., 2015) and are at higher risk of dropping-out (Kwiek & Szymula, 2024).

Looking at the Italian context, multivariate regression-based studies using administrative data on the academic population indicate that the likelihood for women to be promoted remains lower than that of men with equal characteristics, including scientific productivity, both for a position of associate professor (De Paola et al., 2018; Filandri & Pasqua, 2021) and full professor (De Paola et al., 2018; Filandri & Pasqua, 2021; Marini & Meschitti, 2018; Meschitti & Marini, 2023). Similar results come from natural experimental studies based on real recruitment procedures (Checchi et al., 2019; De Paola & Scoppa, 2015). At the same time, both Abramo et al. (2016) and Gërxhani et al. (2023) suggest a better picture, the former coming up with no gender bias in 2008 recruitment procedures for associate professors, the latter pointing out—on the base of a lab experiment—to a gender bias in recruitment in the field of economics, but not in the humanities nor in social sciences.

Coming back to observational studies conducted in Italy, they are all based on ministerial open micro-data which include information, by year, on assistant, associate, and full professors but not on post-docs (see: https://cercauniversita.mur.gov.it/). This explains the fact that all these studies focus on the intermediate and final steps of the academic ladder—that is on the transition from assistant to associate and from associate to full—but not on the early-ones, that is on the transition from post-doc to assistant professor (De Paola et al., 2018; Filandri & Pasqua, 2021; Marini & Meschitti, 2018; Meschitti & Marini, 2023). This gap is partially offset by a second stream of research based on survey data on doctorates graduates which are periodically collected by the National Institute of Statistics (in Italian: ISTAT) with the aim of tracking their work outcomes. For example, by analyzing the 2014 wave, Bozzon and colleagues find that female doctorates are less likely to hold a *stable* position in academia than their male counterparts (Bozzon et al., 2017). However, they do not disentangle among the different types of existing position in academia. By using three waves of the same survey (2010, 2014 and 2018), Carriero et al. (2024) find that female PhD are more likely than men to be out of employment or employed in government and non-profit jobs. However, no specific analysis on the academic sector has been done. Carriero and Naldini (2022)’s paper takes this direction by going down to a greater level of detail: analyzing the 2014 and 2018 waves, the two authors shows that female doctorates do not experience an adjusted disadvantage in the probability to become post-doc while they do in the probability to become assistant professor. However, no distinction between the different types of contracts existing in Italy for assistant professors is made, especially between the “pre-reform,” (tenured and with an open-ended contract) assistant professor and the two “post-reform,” short-term, assistant professors (see next paragraph for further details). But most especially, their analyses are not able to measure the transition from the post-doc to the assistant professor, as long as it is based on a survey which asks PhD holders to declare whether they are—four and six years from PhD completion—either post-doc *or* assistant professor, without allowing to use the position of post-doc as reference category. Up to now, this specific step has been investigated at a descriptive level only (Gaiaschi & Musumeci, 2020; Picardi, 2019). By accessing non open ministerial micro-data on post-docs across time, this study makes it possible to fill this gap.

Besides the variation of the female gap in promotion along the hierarchy, the literature has focused on its explanations. The contributions on this issue are heterogeneous and suggest how explanatory factors are multi-dimensional, being the gap the combined result of factors playing at micro, meso, and macro levels.

The micro level includes individual factors, both on the supply-side and the demand-side. Supply-side factors concern the differences in characteristics between female and male academics, for example in care responsibilities and scientific productivity. On the first point, that of care responsibilities, many studies conducted on academia and science point out to the persistence of a maternity penalty on productivity and promotion (e.g. Hunter & Leahey, 2010; Misra et al., 2012; Mairesse & Pezzoni, 2015; Lawson et al., 2021). Other studies, however, show more nuanced results, with some suggesting that female self-selection in high-skilled professions is likely to neutralize the penalty (Gaiaschi, 2021) and others calling for the importance of looking at the role of the family configuration (number and age of the children, role of the partner) in moderating women’s disadvantage (Fox, 2005; Zippel, 2017). As for the second point, that of gender differences in scientific productivity, the literature generally indicates that women publish less than men (e.g.: Abramo et al., 2021; De Paola & Scoppa, 2015; Huang et al., 2020). On the other hand, the gap narrows when taking in consideration differences in career lengths (Huang et al., 2020) and periods of parental leave (Mairesse & Pezzoni, 2015). Moreover, productivity relies on factors—such as access to networks, the number of co-authors, the size of the research group, and the distribution of financial resources—which are not gender neutral. As such, women’s “lower” scientific performance should be interpreted more as a systemic, rather than an individual, outcome (Gaiaschi, 2022). Additional factors related to the supply-side concern gender differences in mobility and international collaborations (Bozeman & Gaughan, 2011), in financial resources (Ceci & Williams, 2011) and self-promotion: on this last point, studies based on Italy suggest that women are less likely to apply for the National Scientific Qualification, an essential pre-requisite to become professor (De Paola et al., 2017). Finally, on the micro level of the demand-side, gender biases can bring employers and recruiters to prefer men over women, and this can translate into favoritism towards men during recruitment and promotion processes. On this respect, a recent strand of literature has shown how female candidates are often evaluated by recruiters as less competent and capable than men not matter equal levels of productivity and other co-founders, thus suggesting the role of stereotypes in producing inequalities (Bagues et al., 2017; Checchi et al., 2019; De Paola & Scoppa, 2015; Reuben et al., 2014).

The meso level refers to the role of the context in shaping inequalities. This includes the persistence of gender inequality practices in organizations which are at the base of the construction and evaluation of excellence, which systematically generate disadvantages for women (Van den Brink & Benschop, 2011). More structural contributions have focused on networks and homophily, suggesting that men benefit from their higher social capital (Araújo & Fontainha, 2017), on women’s higher concentration in service and teaching (Heijstra et al., 2017), as well as on the role of the scientific field in moderating the gender gap. On this point, studies generally suggest, with few exceptions (e.g. Weisshaar, 2017), that women face fewer obstacles in the social sciences and humanities (SSH) compared to the STEMM (science, technology, engineering, mathematics, and medicine) (Webber and González Canché, 2018; Durodoye et al., 2020; Carriero & Naldini, 2022). Finally, the gender composition of the commissions for recruitment and promotion may also play a role. The evidence on this point is mixed, with some studies pointing out to an amelioration of female opportunities when the commission is gender-balanced (van den Brink, 2010; De Paola & Scoppa, 2015) and others suggesting a deterioration (Duguid, 2011; Bagues et al., 2017).

As for the macro factors, they refer to the broader institutional context, including the structure of the labor market and university policies. On this respect, a few contributions have focused on the relationship between academic and non-academic opportunities, suggesting how women’s retention in academia may also depends on men’s opting out towards better remunerated sectors (Bataille et al., 2017). Other studies have investigated the gendered implications of the recent market-based reforms of university systems. Inspired by the “meritocratic ideal” and paralleled by the cuts in the funds for higher education, such reforms have fostered discourses on excellence emphasizing hyper-productivity while increasing work precarity. On this point, several studies have suggested that such transformations are likely to worsen women’s situation in academia (Deem, 2009; Van den Brink & Benschop, 2011; Lund, 2015), while others are less clear-cut, pointing out to a more complex and ambivalent picture (Ferree & Zippel, 2015; Gaiaschi, 2023).

## The Italian University system and its career ladder

Italy comprises 89 universities—among which 61 public, 20 private, and 8 “special university institutes”—with around 70,000 among post-doc, assistant, associate, and full professors.^2^ Since 2010, careers are regulated by the 240/2010 Law, also called “the Gelmini reform,” which has brought three main changes: (1) the introduction of the “National Scientific Qualification” (NSQ), a national evaluation process managed by the Ministry of University and Research (MUR) awarding a qualification based on standard metrics of individual performance that academics need to hold when applying for the positions of associate and full professors^3^; (2) an increased autonomy for single universities which are free to open up a recruitment or promotion procedure—once regulated at central level; and (3) the restructuring of the assistant professor (from now on: AP) position with the replacement of the old tenured—and with an open-ended contract—AP with two new types of position, both with a short-term contract: a junior non-tenured and a senior tenure-track or “quasi” tenured AP.

Such changes occurred in the frame of a wider process of transformation of the Italian academic system based on market-based principles which occurred at least since the mid-2000 s. The key element of these changes has been the adoption of performance indicators aimed at more efficiently allocating governmental funding to performing universities. Examples include the “Research Quality Assessment” (from now on: RQA), in Italian Valutazione della Qualità della Ricerca (VQR), and the 2017 and 2023 “Department of Excellence” rankings. The RQA evaluates the quality of research outputs provided by Universities, and it is conducted every four years, since 2011, by the National Agency of Evaluation of Universities and Research (in Italian “Agenzia Nazionale di Valutazione dell’Università e della Ricerca” or ANVUR), which grants part of the ordinary annual governmental funds to high-score universities on the basis of their performance. Moreover, the 2017 (first edition) and 2023 (second edition) “Department of Excellence” ranking has awarded single departments with extra research funds on the base of a national competitive call for projects.

Such evaluation-based practices occurred in parallel with the cuts in the funds for higher education in place for nearly a decade which have prevented universities—until 2017—from fully replacing retiring professors with newly appointed ones. As a consequence, the number of unstable contracts, including contract professors and post-doc positions, has risen (De Angelis & Grüning, 2020; Gaiaschi & Musumeci, 2020).

**Fig. 1:**
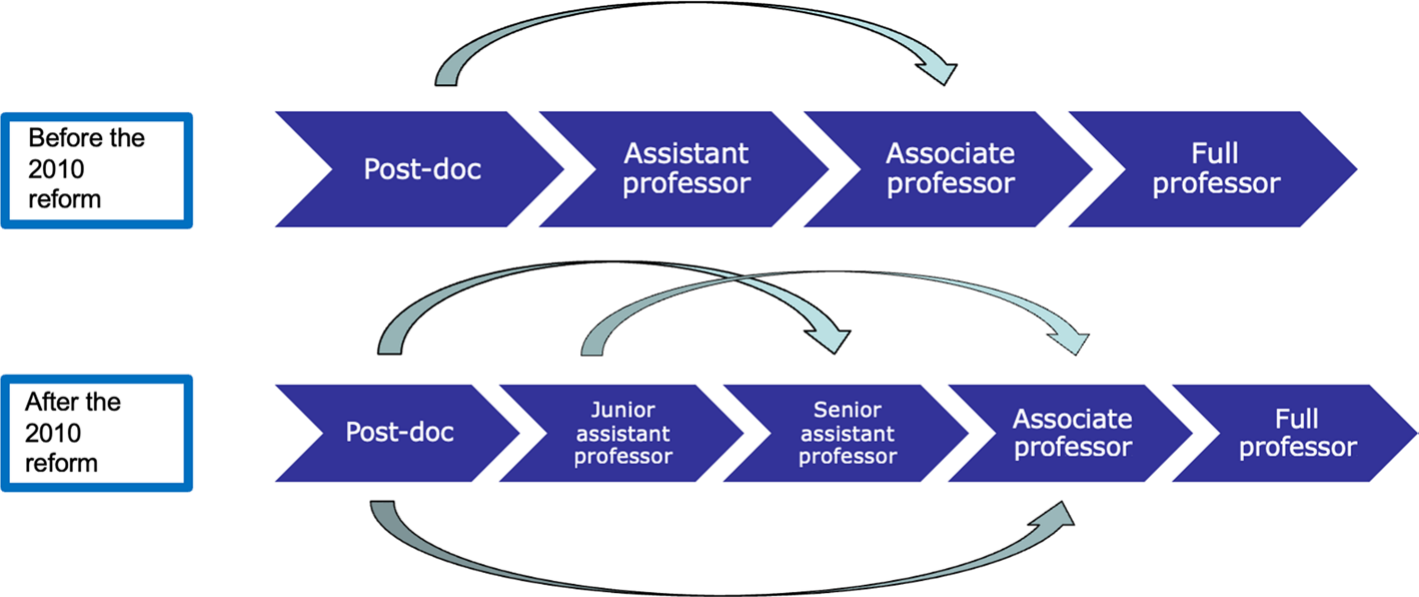
The Italian academic career track

Today, the typical academic track in Italy is characterized by a long period of post-doc fellowships which are often fragmented in contracts of different duration. After the post-doc phase, the “natural” career track foresees the transition to the “junior” (non tenured) AP, a three-year short-term contract which can be extended for further two years. Once the junior AP is finished, the “senior” (tenure-track) AP, which also lasts three years, represents the next step. In practice, things are much more complicated and steps can easily be skipped: for example, a post-doc fellow can directly become a senior AP or, even if this is extremely rare, an associate professor. A junior AP can skip the senior AP phase and become associate professor. As a consequence, the academic career track can be very heterogeneous from one person to another, as illustrated by Fig. 1.

Recently, the law n. 79/2022 has introduced a further change in early-career phases, by replacing the two types of APs with a unique position, that of “tenure-track” AP. This new type of contract has a maximum length of six years and, if the researcher has obtained the national scientific qualification, is automatically transformed—after a positive evaluation of the department—to an associate professor’s position. Being comprised between 2010 and 2020, the data used for this article do not include this new type of position, whose very first contracts were implemented only in 2023.

## Research objectives and hypotheses

This paper analyzes the gender recruitment gap among academics in Italy. The aim of this research is twofold: to measure whether women experience an adjusted disadvantage in the transition from the post-doc to the AP position and, if it is so, to identify the explanatory factors at the base of such disadvantage. With respect to the first issue, the gender difference in the probability of becoming AP will be measured in first place, without making a difference between junior and senior contracts: this will allow to measure the likelihood to become AP, regardless of the outcome (Model 1). Moreover, more detailed models will be presented in order to take in consideration all the possible types of career transition: from the post-doc to junior AP (Model 2), from the post-doc to the senior AP (Model 3), and from the junior to the senior AP (Model 4).

After analyzing the gender recruitment gap, its explanatory factors will be investigated by focusing on contextual characteristics, namely the scientific field in which academics work, the rate of female full professors in the sub-field, and the department’s productivity. More specifically, the aim of the analyses will be to measure the variation of the gender gap across different fields, levels of feminization of decision-making positions, and organizational performance.

With respect to the scientific field, studies comparing the variation of the adjusted gender gap according to the field are few (Weisshaar, 2017; Webber and González Canché, 2018, Carriero & Naldini, 2022) and many of them focus on a single field or groups of fields, mostly in the STEMM (Bozzon et al., 2017; Gërxhani et al., 2023). For this reason, scholars have called for a more granular approach to examine between-discipline differences (Durodoye et al., 2020). To fill this gap, the gender differential in recruitment has been measured across scientific fields. Consistently with previous studies on this point (Webber & González Canché, 2018; Durodoye et al., 2020; Carriero & Naldini, 2022), I expect the gap to be smaller in the SSH compared to the STEMM (Hypothesis 1).

The scientific field is a proxy of the contribution of feminization processes in easing inequalities. A better, and more direct, measure is the rate of feminization itself. The number of women, and so the idea that the attainment of a “critical mass” can make a change, especially when it occurs in decision-making positions, has been a long-debated issue since Moss Kanter (1977) work on female top managers. According to her theory, when women surpass a percentage that the author approximates to around 35%, changes in the culture of the group may occur. The critical mass theory has been tested relatively to the academic sector by looking, for example, at the effect of the gender composition of the commissions (for recruitment and promotion) on women’s opportunities (to be recruited and promoted): on this point, however, the results are rather mixed with some studying arguing a benefit, for women, from gender-balanced commissions (van den Brink, 2010; De Paola & Scoppa, 2015) and others suggesting a deterioration (Duguid, 2011; Bagues et al., 2017). Others show mixed results: Zinovyeva and Bagues (2011) find a positive effect for the competitions for full professors but not for associate professors; Abramo et al. (2015) find a benefit when the president of the commission is a woman, but not when the commission is gender balanced. Working on a dataset similar to the one used for this article, Filandri and Pasqua (2021) look at the contribution of the number of female full professors by sub-field on the gender gap. Face to the lack of data on recruitments, focusing on the feminization of sub-field may be considered a good proxy of the effect of feminization processes on women’s opportunities given that sub-fields (in Italian: SDS, see more on this point in the next section) represent the power locus where promotions are controlled, especially when it comes to obtaining the National Scientific Qualification which is necessary to access tenure-track positions. Filandri and Pasqua’s analyses come up to a positive association between feminization and women’s promotion. Their analyses concern the transitions to associate and to full professors. The same analyses have been run here by focusing on the transition from the post-doc to the AP. Thus, I expect that a growing number of women full professors in the sub-field may reduce the gender gap (Hypothesis 2).

In the same article, Filandri and Pasqua (2021) use the size of the university as a proxy of financial resources available for promotion in order to understand whether richer institutions offer better opportunities for female candidates. The dataset used in this article includes two more direct measures of resources which are both based on organizational performance: the score that Universities have obtained, by field, in the 2011–2014 and 2015–2019 waves of the RQA, and the award that Departments have obtained (or not) in 2017 as “departments of excellence” in the homonymous ranking. Both are based on the productivity of the faculty plus—for the “departments of excellence” award—a research development plan proposed at the department’s level. This 2017 department of excellence ranking particularly interesting because the awarded departments receive extra and generous funds, part of which are explicitly used for recruitment. For this reason, it appeared as an ideal measure to test the relation between financial resources and gender equality. Thus, I expect that departments having been awarded as departments of excellence to show a smaller gender gap compared to non-awarded departments (Hypothesis 3).

## The dataset

The analyses presented in this work are based on an original dataset on the academic population working in Italy from 2010 to 2020 which is the result of the harmonization of multiple sources including (1) administrative panel micro data on Italian academics and (2) administrative panel micro data on the NSQ. Both datasets are anonymous and provided by the MUR within the frame of the WIRED project (grant number 898507). Furthermore, (3) webscrapped (public) data on organizational performance have been also collected.

The first dataset includes demographic (gender, year, and state of birth) and work information such as the rank held by the individual, the scientific field (in Italian “area scientifica”) and sub-field (in Italian “settore scientifico disciplinare” or SDS), the University, and the department in which she/he works. The MUR classification comprises 14 “areas”,^4^ each of them includes manifold “SDS” for a total number of 373.

The second dataset includes information related to the first four waves (2012, 2013, 2016, 2018) of the National Scientific Qualification, that is all the applications sent by each candidate (who can apply and obtain more than one NSQ in different sub-fields) with the related “wave” (“tornata”) and “sub-wave” (“quadrimestre”), the position (whether it is for associate or full professor), the scientific sub-field (“settore concorsuale”), the result (whether he/she has succeeded or failed), the date of obtention, and the three indicators of the candidate’s scientific productivity—covering the ten years preceding the evaluation—with the three relative thresholds, that is a minimum standard value for each indicator according to the sub-field in which the candidate applies.^5^ For the purpose of this study, whether the individual has applied or not to at least to one habilitation for associate professor, whether he/she has obtained it, and the scores obtained for each of the three indicators—an information that refers both to successful and failed candidates—have been retained. In case of multiple habilitations, the habilitation related to the last sub-field in which the individual has been observed has been used. As for the three productivity indicators, they were centered around their thresholds in order to standardize them across disciplines. Afterward, their mean was computed so to obtain one single metric—named “NSQ score”.

With respect to the information provided by the NSQ dataset, an important consideration should be made. Candidates are granted (or not) with the NSQ on the base of an evaluation of a commission which is periodically appointed by the Ministry (and specific to the sub-field in which the candidate apply), based both on qualitative and quantitative criteria. The qualitative criteria include, for example, the list of publications and other scientific titles, such as the coordination of research projects, patents, visiting positions abroad, teaching in PhD courses, and so on. Each commission must also provide an opinion on a limited set of publications submitted by each applicant (Abramo & D’Angelo, 2015). The quantitative criteria are based on three numeric indicators of impact and productivity. For the so-called bibliometric fields—that is the STEMM plus psychology—the three indicators are based on bibliometric criteria, including citations, while this is not the case for the SSH (see footnote n.5 for more details). As such, the "NSQ score"—as a proxy of individual productivity—appears to be more reliable for bibliometric fields than non-bibliometric fields. On the other hand, existing web-scrapped measures of the individual productivity in the SSH—based on search engines such as Scopus or Google Scholars—are not exempts from the risk of being biased (Aksnes & Sivertsen, 2019). Moreover, one should consider that the NSQ indicators allow to compare the productivity between the STEMM and the SSH, on the ground that thresholds represent their common denominator (Meschitti & Marini, 2023). For this reason, and notwithstanding the caution needed in this case, the NSQ score has been used as a proxy of individual productivity. As for the variable indicating whether the individual holds (or not) the NSQ ("NSQ y/n"), as said, the overall evaluation of the commission, and so the decision to grant or not the NSQ, relies not only on the quantitative but also on the qualitative profile of the candidates. As such, as pointed out by several scholars, it is not exempt from subjective biases in the evaluation, networking mechanisms, and dynamics of discrimination and favoritism (Abramo & D’Angelo, 2015; Marzolla, 2016; Jappelli et al., 2017). As a consequence, it should not be interpreted as a proxy of individual productivity but, rather, as a mere title. Its inclusion in the models is, in any case, extremely important as it represents a criterium which is often required in the public selections for a position of tenure-track assistant professors.

Together with administrative data on the academic population, including information on the NSQ, data on organizational performance have been webscrapped: they include the 2017 ranking of the “Departments of Excellence” and the 2011–2014 and 2015–2019 waves of the Research Quality Assessment (RQA). The first database indicates which departments have been granted “department of excellence” on the base of their RQA evaluation and a development plan in 2017. The second indicates the evaluation that universities and departments have obtained in relation to the different scientific fields and subfields in terms of score and ranking based on the number and the quality of scientific products related to the years 2011–2014 for the first wave and 2015–2019 for the second wave. For our analyses, the scores that each university has received in relation to the different scientific fields were used.

Given the purpose of the study, the dataset used for the regression analysis was restricted to the years 2010–2020 and to post-docs (in Italian “assegnisti di ricerca” or AR) and post-reform assistant professors, both junior and senior (in Italian RTDa and RTDb respectively), while excluding the post-docs who have become pre-reform assistant professor.^6^ Further choices have been made in order to reduce other possible biases like, e.g., excluding from the dataset those (very rare) post-doc who have skipped the assistant professor step and directly become associate professor—610 individuals of whom, it is worth to mention, more men (392) than women (218)—and those who have reversed career track (i.e., individuals who re-became post-doc after being AP). The final sample comprises 64,736 individuals—among which 32,992 women and 31,744 men, equal to 189,678 observations. Considering missing data, which are mostly related to the NSQ score, something that only those how applied (regardless of the result) to the NSQ have, the number of individuals considered in the broader model M1 (see further down for details) sharply decreases to 17,534 individuals, equal to 79,110 observations.

## Models and measures

In order to measure the gender disadvantage in the transition from the post-doc to the AP position, three-level random intercept linear probability models (LPM) have been used. The multi-level approach is essential to account for the nested nature of the data with years (level 1 unit) nested in individuals (level 2 unit) and individuals nested in universities (level 3 unit). Data, however, are not perfectly hierarchical, as individuals can switch from one university to another during their academic life. As a consequence, the possibility of multiple memberships or crossed-random effects (Rabe-Hesketh and Skrondal 2012), with individuals observed in more than one university during the ten years of observations, has been taken into account by adding crossed-random intercepts for universities (Rabe-Hesketh and Skrondal 2012). As for the choice of using linear probability models, their coefficients are almost identical to the average marginal effects of the logit models, with the advantage that the interpretation of the coefficients is simpler (von Hippel, 2015, 2017). Different analyses have been replicated according to the codification of the dependent variable (DV), a dummy indicating the position of the respondent. Model 1 tests the transition from the post-doc to the AP position, as the DV assigns 0 if the observation is a post-doc and 1 if he/she is AP, no matter the type. Models 2–4 go more in detail and track the transition from the post-doc (0) to the junior AP (1) in M2, from the post-doc (0) to the senior AP (1) in M3 and from the junior (1) to the senior (2) AP in M4. As such, they are based on different sub-groups of the population. The equation for the three-level random (or “varying”) intercept LPM is the following:

$${Y}_{tiu}={\beta }_{0}+{\beta }_{i}gender++\sum {\beta }_{i}{X}_{i}+{v}_{u}+{u}_{iu}+{\epsilon }_{tiu}$$

where *Y* is the dependent variable, *β*_*0*_ is the intercept, that is the mean response across universities, *β*_*i*_*gender* refers to the coefficient of the independent variable (from now on: IV), *∑β*_*i*_*X*_*i*_ to the coefficients of the control variables, *v*_*u*_ is the crossed random intercept for the three-level unit, that is the effect of university *u* (super-cluster random effect), *u*_*iu*_ is the random intercept for the two level units, that is the effect for individual *i* (cluster random effect), and *ϵ*_*tiu*_ is the residual error term which is specific to each occasion (year) *t* for each individual *i* in university u.

The results of the analyses are displayed in Table 4.^7^ The table shows the four full models M1–M4 with all the predictors, including the IV “gender” (1 = woman; 0 = man), which should be interpreted as the female coefficient to promotion, with the remaining controls. Controls include a time variable named “year” indicating the year of observation as well as individual (micro) and contextual (meso) characteristics. Individual characteristics refer to the individual; contextual factors refer to higher-level units in which the individual is nested. Individual-level variables include an interval variable for age, a dummy variable for the nationality (1 = Italian; 0 = all others) based on the state of birth (variable “Italian”), a dummy variable for having obtained the NSQ (1) or not (0) (“NSQ y/n”) and a continuous variable for the NSQ standardized score (“NSQ score”). Moreover, a three-item variable for the scientific field recodified according to the ERC sectors (0 = physical sciences and engineering or PE; 1 = life sciences or LS; 2 = social sciences and humanities or SH) was added. Contextual-level variables include an interval variable indicating the percentage of female full professor by sub-field ("Female full prof by sub-field"), an interval variable indicating the number of academics working in the university in which the individual is observed (“University size”), a dummy variable indicating whether the department in which she/he works has been awarded in 2017 as a department of excellence (1) or not (0) (“2017 excellence ranking”) and a continuous variable indicating the score that the university has obtained for each scientific field in the two ministerial RQA (“RQA score”). Given that the criteria used for the evaluation have slightly changed across the two waves, a dummy variable indicating whether the score has been obtained in the first (0) or in the second wave (1) (“RQA wave”) has been added as control. Time-varying variables include, beside year and age, the qualification (“NSQ”), the percentage of female full professors by sub-field, the university size, the 2017 excellence ranking, and the RQA score.

Table A5 in the Appendix compares the female coefficients of the adjusted models reported in Table 4 with the female coefficient of the unadjusted models. Unadjusted models include only the IV “gender” and the time variable “year.” Table A6 use the MUR classification of scientific fields based on 14 “areas” instead of the three-items of the ERC classification use in Table 4.

**Fig. 2:**
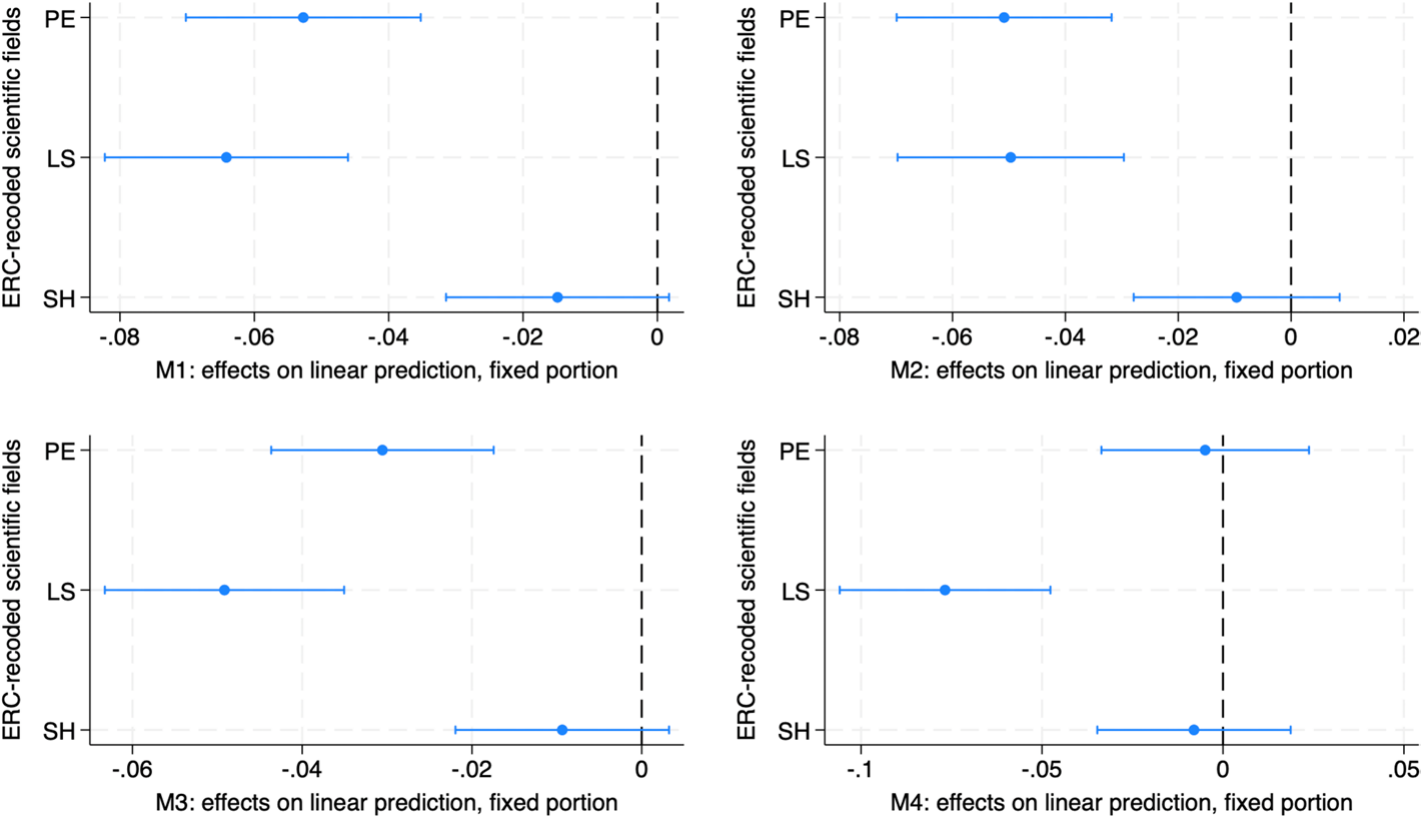
The scientific field (ERC codification): gender differences in predicted margins

**Fig. 3:**
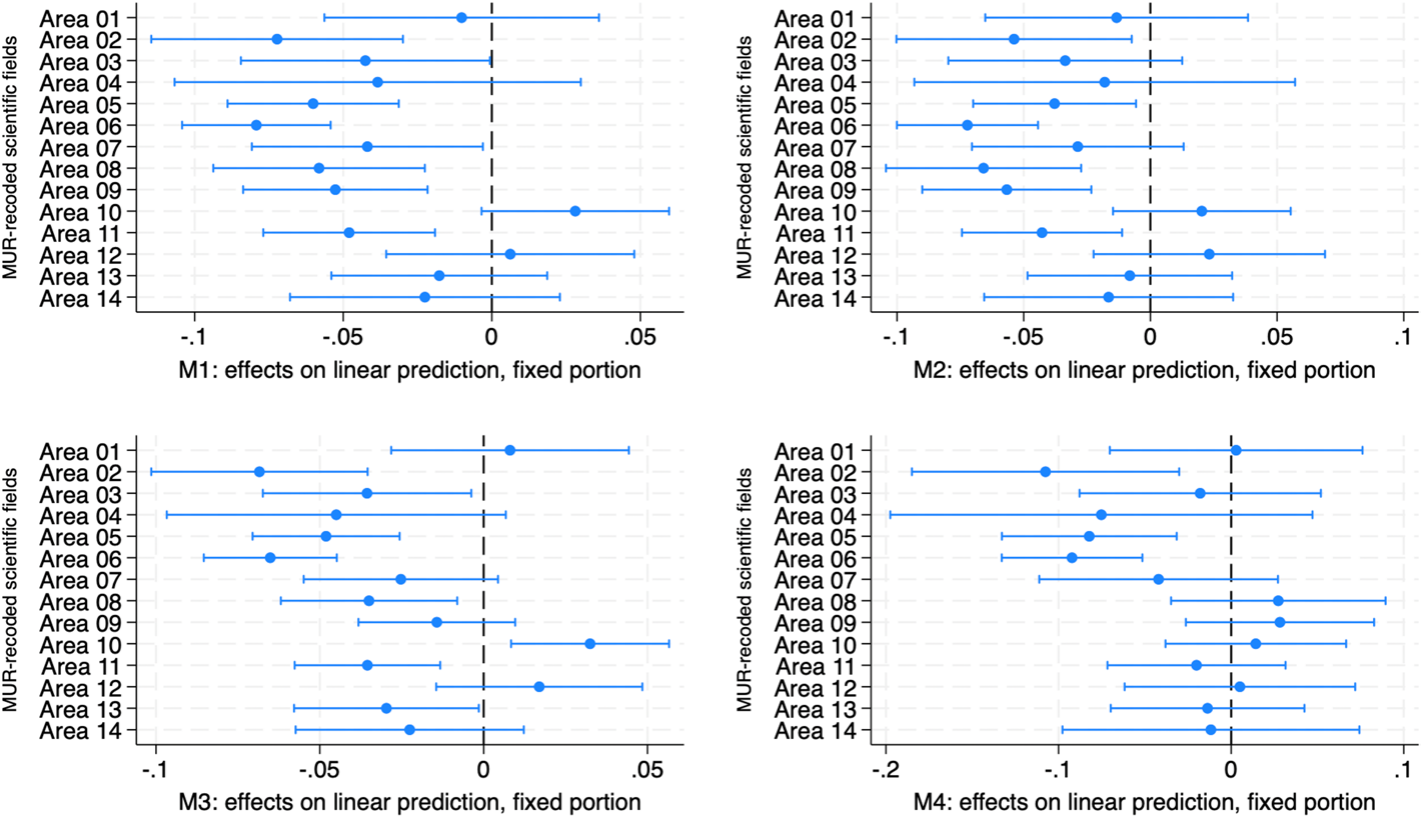
The scientific field (MUR codification): gender differences in predicted margins

**Fig. 4:**
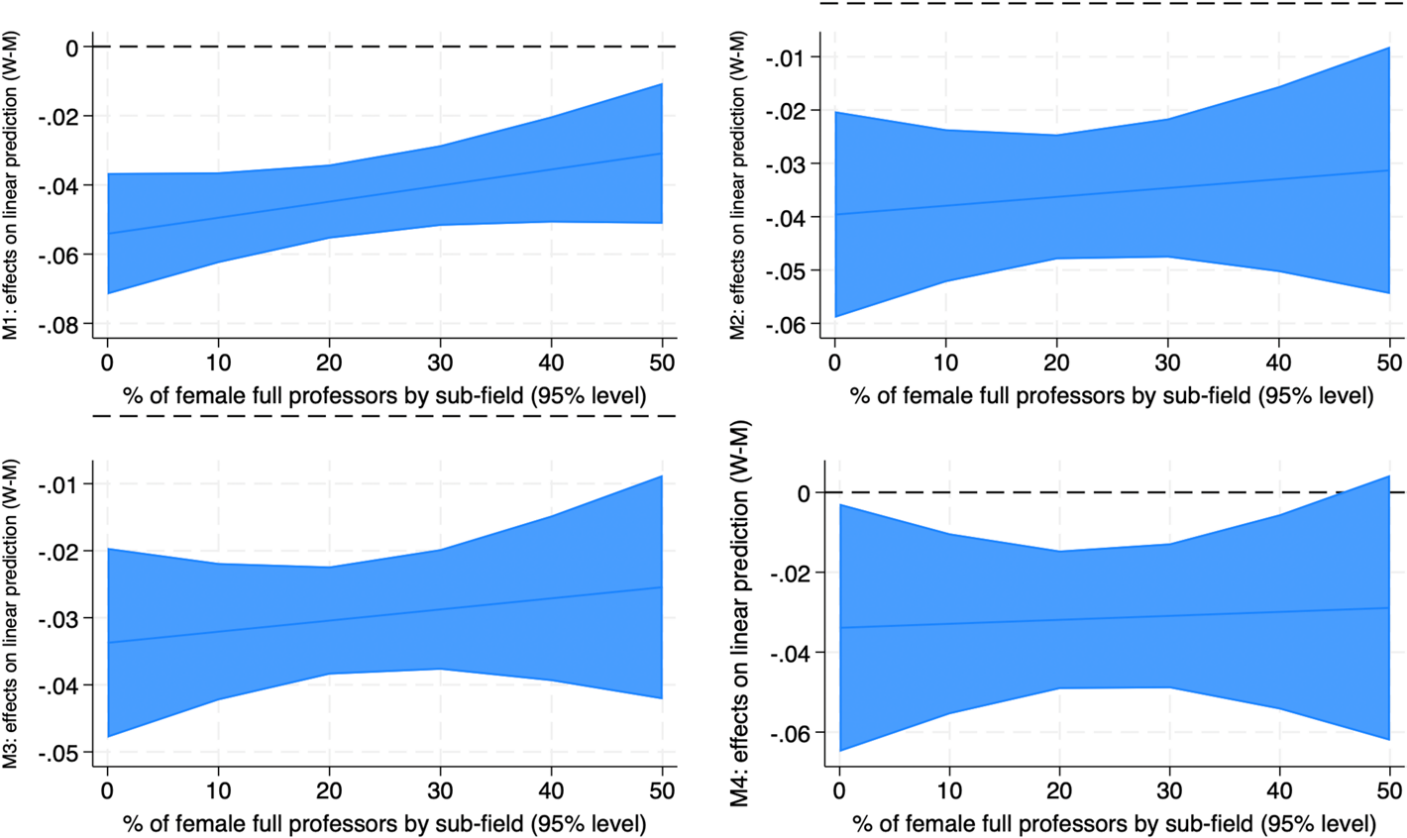
Share of female full professors by sub-field: gender differences in predicted margins

Once the female disadvantage in recruitment has been measured, its factors were investigated by means of interaction models which make it possible to measure the different “returns” in terms of recruitment, for men and women, of a specific characteristics. At this regard, two-way interaction terms between the modifying variable gender and three independent variables (the scientific field, the feminization of full professorship by sub-field and the 2017 excellence ranking) have been added in the models. Once added, the predicted margins or the partial changes in the predicted margins (marginal effects) have been computed. Figures 2, 3, and 4 and Table 5 summarize the results of these analyses (full interaction models available upon request).

## Findings: descriptives

Table 1 provides an overall picture of the population under analysis. As mentioned, the dataset includes 189,678 observations, equal to 64,736 individuals. Given the panel nature of the dataset, and so the possibility, for the same individual, to move across the career ladder and, as a consequence, to be observed in more than one position across time, the number of individuals *per position* is slightly higher than the total number of individuals: 76,329, among which 60,408 post-docs. Out of the 60,408 post-docs, however, the larger portion, more specifically 86%, didn’t reach the position of assistant professor, either junior or senior, with a slight (but statistically significant) majority of women (26,261) over men (25,673) (see Table A2 in the Appendix). These cases can be interpreted, for the most part, as individuals who have exited the academic career track, but also, as individuals who have gone abroad or, more simply, as right-censored cases, that is post-docs who have become assistant professor after 2020. Table A3 in the appendix provides further, detailed, descriptive information on the population under analysis by showing the distribution of men and women by position and year.

**Table 1:** Observations and individuals by position

	Observations		Individuals	
	Freq	%	Freq	%
Post-doc	148,358	78.22	60,408	93.31–1
Junior AP	25,524	13.46	9,375	14.48
Senior AP	15,796	8.33	6,546	10.11
Total	189,678	100.00	76,329	117.91

(*n* = 64,736)

Table 2 shows the gender distribution across positions and scientific fields - based both on the ERC and MUR classifications (move note n.9) - on the pooled dataset, -that is on observations. As expected, the post-doc phase is rather gender balanced, with 51% of women and 49% of men. However, the female rate decreases in the following steps, with 44% of women among the junior assistant professor and 41% among the senior ones, against 56% and 59%, respectively, of the men. Significant differences also concern the scientific field. Female post-docs and assistant professors are particularly under-represented in the PE, where they make 35% of the observations, against 65% of the men, driven by—when looking at the more detailed MUR classification—the field of mathematics and informatics and the field of engineering trades and manufacturing, which are the least feminized fields with only 27% of women in both cases. At the same time, the LS are even more feminized than the SH, with 64% of the women in the former and 54% in the latter. The MUR classification suggests that this is mostly due to the fields of the biological and the medical sciences (which make 80% of the observations in the LS), where women represent 65% and 67% respectively of the total population. These numbers could be quite surprising but one must not forget that the sample analyzed include post-docs and post-reform AP only, and so the youngest generations, among which the feminization of the life sciences is stronger. In fact, when considering the broader dataset, comprising pre-reform assistant professors, associate, and full professors, the rate of women in such fields decreases, especially in the medical sciences, where women represent only 13.4% of full professors, thus suggesting a strong phenomenon of leaky pipeline (see Table A4 in the appendix on this point).

**Table 2:** Gender differences by position and field (observations), 2010–2020

	Men	Women	*p*-value
**Position (%)**			
Post-doc	48.9	51.1	0.000
Junior assistant professor	56.1	43.9	0.000
Senior assistant professor	59.3	40.7	0.000
**Scientific field**	Men	Women	*p*-value
**ERC classification (%)**			
PE (Physical sciences and engineering)	64.91	35.09	0.000
LS (Life sciences)	36.04	63.96	0.000
SH (Social sciences and humanities)	45.86	54.14	0.000
**MUR classification (%)**			
01-Mathematics and informatics	72.48	27.52	0.000
02-Physical sciences	69.97	30.03	0.000
03-Chemical sciences	44.99	55.01	0.000
04-Earth sciences	59.76	40.24	0.000
05-Biological sciences	35.20	64.80	0.000
06-Medical sciences	33.24	66.76	0.000
07-Agriculture and veterinary	44.35	55.65	0.000
08-Architecture and construction	53.94	46.06	0.000
09-Engineering trades and manufacturing	73.19	26.81	0.000
10-Archeology, languages and arts	41.59	58.41	0.000
11-History, philosophy, psychology, education	43.18	56.82	0.000
12-Law	50.19	49.81	0.001
13-Business, administration, and statistics	48.78	51.22	0.000
14-Political and social sciences	48.40	51.60	0.000

*p*-value of chi2 test for the analysis of variance

Number of observations: 189,678

Table 3 provides information on the National Scientific Qualification (NSQ).^8^ Out of the 64,736 individuals observed, the majority, 72%, did not apply, 5.5% applied but failed, while 22.5% succeeded. When looking at gender differences, the table shows that fewer women than men have obtained the NSQ (19.6% vs 25.4%), but this is mainly due to the fact that more women than men did not apply (74.7% vs 69.4%), while no relevant difference emerges in terms of failure (5.7% of the women vs 5.3% of the men), in line with previous contributions (De Paola et al., 2017). At the same time, when they apply for the qualification, women report a lower NSQ score. The comparison of the mean values by gender run on both failed and successful candidates among the bibliometric population shows that women report a mean NSQ score of 120.8 vs 136.8 of the men (analyses available upon request). The gender gap does not change much if considering only those who obtained the NSQ (134.3 vs 148.5). Women’s lower score could be due to the fact that they are less “productive”^9^ and/or to gender biases in evaluation, as some scholars suggest (Krawczyk & Smyk, 2016; Jappelli et al., 2017).

**Table 3:** Gender differences in the NSQ (individuals), 2010–2020

	Men	Women	Total
Not applied	22,884	23,704	46,588
%	69.36	74.67	71.97
Applied and not obtained	1,741	1,807	3,548
%	5.28	5.69	5.48
Applied and obtained	8,367	6,233	14,600
%	25.36	19.64	22.55
Total	32,992	31,744	64,736
%	100	100	100

## Findings: models

Table 4 reports the full models according to four different specifications of the dependent variable. All in all, results suggest that, controlling for gender differences in observable characteristics, the disadvantage for women is in general, quite low, ranging around − 3/− 4% according to the specification.

**Table 4:** Random intercept multi-level linear probability models on the position

	M1	M2	M3	M4
	Post-doc >	Post-doc >	Post-doc >	Junior AP >
	AP (j + s)	Junior AP	Senior AP	Senior AP
Year	0.0699***	0.0674***	0.0378***	0.0847***
	(0.000966)	(0.00106)	(0.000891)	(0.00165)
**Gender**	− 0.0422***	− 0.0353***	− 0.0281***	− 0.0284***
	(0.00531)	(0.00584)	(0.00400)	(0.00840)
Age	0.0176***	0.0160***	0.0134***	0.0228***
	(0.000477)	(0.000530)	(0.000370)	(0.000789)
Italian	0.0550***	0.0633***	0.0355***	0.0422
	(0.0139)	(0.0154)	(0.0105)	(0.0231)
NSQ (y/n)	0.153***	0.122***	0.420***	0.192***
	(0.00349)	(0.00407)	(0.00351)	(0.00505)
NSQ (score)	0.0000763***	0.0000643***	0.0000631***	0.0000601***
	(0.0000102)	(0.0000112)	(0.00000769)	(0.0000150)
ERC field: PE	0	0	0	0
	(.)	(.)	(.)	(.)
ERC field: LS	− 0.00262	0.00758	− 0.0155**	− 0.0308**
	(0.00660)	(0.00729)	(0.00514)	(0.0110)
ERC field: SH	− 0.0274***	− 0.0402***	− 0.0148**	0.0409***
	(0.00705)	(0.00784)	(0.00557)	(0.0118)
Female full prof by sub-field	0.000311*	0.000334*	0.000117	− 0.000225
	(0.000139)	(0.000158)	(0.000119)	(0.000238)
University size	0.0000775***	0.000105***	0.0000674***	0.0000409**
	(0.0000102)	(0.0000120)	(0.00000840)	(0.0000146)
2017 ranking of excellence	0.00427	− 0.00410	0.00824	0.0575***
	(0.00491)	(0.00603)	(0.00456)	(0.00786)
RQA score	0.0958***	0.0644**	0.0842***	0.193***
	(0.0211)	(0.0232)	(0.0181)	(0.0412)
RQA wave	− 0.0172***	− 0.0347***	− 0.0147**	− 0.0377***
	(0.00500)	(0.00533)	(0.00467)	(0.00883)
Constant	− 1.523***	− 1.462***	− 1.066***	− 2.267***
	(0.0328)	(0.0379)	(0.0261)	(0.0530)
*N*	79110	63722	58379	36119
*n*	17534	16344	16192	10437
University level variance	0.0234639	0.0368307	0.0094906	0.0210741
SE	(0.0046548)	(0.007188)	(0.0024022)	(0.0041453)
95% CI	0.0159 | 0.0346	0.0251 | 0.0539	0.00578 | 0.0156	0.0143 | 0.0309
Individual level variance	0.0926826	0.1061137	0.0428687	0.1402206
SE	(0.0012771)	(0.001475)	(0.00071)	(0.0025888)
95% CI	0.0902 | 0.0952	0.1032 | 0.1090	0.0415 | 0.0443	0.1352 | 0.1454
Residual variance	0.0761623	0.0717139	0.0491949	0.0752289
SE	(0.0004371)	(0.0004679)	(0.0003436)	(0.000689)
95% CI	0.0753 | 0.0770	0.0708 | 0.0726	0.0485 | 0.0499	0.0739 | 0.0766
*N*	79,110	63,722	58,379	36,119
*n*	17,534	16,344	16,192	10,437

Standard errors in parentheses

* *p* < 0.05, ** *p* < 0.01, *** *p* < 0.001

When looking at the contribution of the remaining predictors on recruitment, holding the National Scientific Qualification for associate professor represents without any doubt the strongest explanatory factor in order to obtain a position of assistant professor, swinging from an effect of around + 12% to + 42% depending on the model, with a particularly strong effect in M3, and so in the transition from the post-doc to the senior assistant professor position. This is not surprising given that the NSQ, when it is not included in the list of titles to hold in the call for application for a position of senior assistant professor (which it is often the case), in any case represents—de facto—an informal requirement to obtain that position. At the same time, the NSQ standardized score—and so the evaluation of the *individual* productivity—has an extremely small positive coefficient, nearly to null. With this respect, what counts more for recruitment is the *organizational* productivity, more specifically the score that the university has obtained for the scientific field in which the individual works within the frame of the Research Quality Assessment procedure (“RQA score”). In this case, the coefficient swings from + 6% to + 19% according to the model. At the same time, the effect of working in a Department which has been awarded as “Department of Excellence” in 2017 is not as clear, with a null effect in M1-M2-M3 and a + 6% in the last model.

Looking at Table A5 in the Appendix, results indicate that there is almost no difference between the unadjusted and the adjusted gender gap, suggesting either that gender differences in observable factors play a marginal role in explaining the gap or that they play a role but in different directions according to the factor (which explains that the overall effect of the controls is null). In order to better understand this point, looking at the variation of the female coefficient once the controls are added once at a time in the models may be useful. By doing so (analyses available upon request), it appears that only two factors have a slight gender effect: age and—at least for M3 and M4—the fact of holding the NSQ. Once added in the model, both of them “moves” the female coefficient but in different directions: the former increases the female disadvantage of around 1–1.5 percentage points (p.p. from now on) depending on the model, the latter decreases it of around 1 p.p. in M3 and M4, while the effect is marginal in the first two models (analyses available upon request). Descriptives suggest that female post-docs are slightly older than male post-docs (33.8 vs 34.5 years old) because, presumably, they become assistant professor later. Paradoxically, the fact of experiencing a discrimination in terms of time of recruitment contributes to reduce the unadjusted gender gap, that is the gap without controlling for age differences between men and women. Likewise, but in the opposite direction, women’s lower likelihood to hold the NSQ (see Table 3) slightly increases the gap in those models—M3 and M4—that measure the transition to tenure-track positions, the ones for which the NSQ is required. This suggests that the fact that fewer women, than men, hold the NSQ, partially explains the unadjusted gender gap in recruitment.

Parallel to the effect—on the gender gap—of *gender differences in characteristics* (like for example in age and NSQ)*,* another important aspect to consider is the *gender difference in the effect of the same characteristics*. To do so, interaction terms have to be added in the models and male and female predicted margins computed. This makes possible not only to measure in which extent a specific factor has different “returns” for men and women, but also to measure the *variation of the gender gap* across different values of this same factor, in this case, the scientific field (hypothesis 1), the rate of feminization of decision-making positions (hypothesis 2), and the organizational performance (hypothesis 3).

The analysis conducted on scientific fields suggests that they play a substantial role in moderating the gender gap in recruitment. Figure 2 shows the marginal effects (more precisely: the average discrete effect), as differences in the male and female predicted margins, computed on the interaction term between gender and the scientific field recoded according to the ERC categories. In all models, women experience a significant disadvantage of around 5–8% in the LS. Surprisingly, the PE—where women are the least represented—report a smaller gender gap, swinging around 3–5% in the first three models while no significant gender gap appears in the fourth. As for the SH, no gender discrimination in this case occurs regardless of the model.

The same analyses were run using the more detailed ministerial (MUR) categorization of the scientific fields (“area”, in Italian).^10^ Figure 3 suggests that the female penalty experienced in the LS seems to be driven by the medical sciences (area 06) where women are around − 6%/− 9% less likely to become AP, followed by the biological sciences (area 05), where the penalty ranges between − 4 and − 8% according to the model. As for the PE, no penalty occurs in mathematics and informatics (area 01). The chemical sciences show a modest penalty (− 3%/− 4%) on M1 and M3 while the earth sciences (area 04) are penalizing in the third model only (and at 90% level). Engineering, trades, and manufacturing (area 09) show a − 5% disadvantage in M1 and M2 while no significant gap occurs in the remaining two models, while architecture and engineering (area 08) show a penalty ranging from − 3 to − 6% in the first three models. Rather, the strongest penalty in the PE occurs in the physical sciences (area 02) where it swings between − 6 and − 10%. Physics, medicine, and biology are the only three fields reporting a negative and significant penalty in M4. As for the SH, no gender gap occurs in the five fields considered expect for the area of history, philosophy, psychology, education in the first three models, where it ranges from − 3 to − 5% and for the area of business, administration, and statistics in the third model (− 3%).

In summary, results show that the LS are the most gender unequal, driven by the medical and the biological sciences. The PE show a smaller penalty, which is mainly due to the physical sciences, while the SH do not report a gender gap. As a consequence, H1—expecting a smaller gender gap in the SSH with respect to the STEMM—should be accepted. However, within the STEMM, a distinction between the hard and the life sciences should be done, with the former being more gender unequal than the latter.

One of the main arguments to explain the variation across scientific fields is their rate of feminization. Traditionally, the literature has suggested that the more a feminized a sector/occupation/field is the more gender equal is. Once the critical mass is reached, women experience an amelioration of opportunities (Moss Kanter, 1977). By simply looking at the percentage of women across the 14 MUR areas both in the whole academic population and among full professors only (Table A4 of the appendix), the support for the critical mass theory is not so straightforward and no clear indication emerges, at least at a descriptive level.

In order to better explore the relation between feminization and opportunities, an interaction term between gender and the percentage of female full professors by sub-field was included in the models. The sub-field ("SDS") was preferred to the wider field ("area") because it's more directly related to career transitions, given that recruitment commissions are SDS-based. The analysis was restricted to the subfields with up to 50% of women full professor given the extremely low number of cases in which they overcome that threshold (Filandri & Pasqua, 2021). Figure 4 shows the marginal effects as differences in the male and female predicted margins of recruitment across different levels of female full professors by sub-field. The results show a marginal benefit from the increasing number of women in the sub-fields in terms of gender gap. The effect is the biggest in M1, where the gap decreases from − 5 to − 3%, while M2 and M3 witness a smaller amelioration, less than one p.p. In M4, the coefficient, which show a rather flat change, becomes non-significant when women overcome the 45% threshold. On the other hand, the confidence interval in this model appears particularly big, due to the low numbers of observations. All in all, the support to the critical mass theory (Moss Kanter, 1977) is not so straightforward: results show a positive but small effect, on recruitments, but further in-depth analysis on this point should be done. Therefore, hypothesis 2—according to which a growing number of women reduces the penalty—should be cautiously accepted.

Together with the feminization of full professorship, resources may have an effect on female recruitment as well. To test this hypothesis, an interaction between gender and the 2017 department of excellence variable has been included in the models. Given that the variable is time-varying, the analyses were run on a sub-sample including the years 2017–2020 only. Table 5 reports the marginal effect of working in a 2017 “department of excellence” for men (first column), women (second column), as well as the differences in male and female predicted margins, and so the gender gap, by type of departments (third column). The results indicate that working in a department of excellence has a negative effect in M2 and a positive effect in M4 for both men and women (with the female penalty in M2 significant at 90% level). This suggests that the surplus of financial resources that departments of excellence have received may have been used more to recruit senior AP than junior AP. The consequences in terms of gender gap are mixed: working in a department of excellence does not ameliorate the disadvantage for female post-docs becoming junior AP (M2), as the gap barely moves. On the contrary, it ameliorates the chances of transition to senior AP, both from the post-doc (M3) and, especially, from the junior AP (M4), where the gender gap for those who work in a 2017 department of excellence fails short. All in all, results mildly suggest that working in a department of excellence ameliorates the chances for women, but only in the transition to senior AP and especially for female junior AP, for whom the penalty actually disappears. As a consequence, hypothesis 4 should be accepted in relation to senior AP but not junior AP.

**Table 5:** Departments of excellence: marginal effects by gender and gender differences in predicted margins

Model		Awarded	Men		Women		W-M	
M1	Post-doc > AP (j + s)	NO	0	(.)	0	(.)	− 0.044***	(0.0072)
		YES	− 0.016	(0.0096)	− 0.007	(0.0109)	− 0.034**	(0.0131)
M2	Post-doc > junior AP	NO	0	(.)	0	(.)	− 0.038***	(0.0095)
		YES	−0.028*	(0.0128)	− 0.026	(0.0144)	− 0.035*	(0.0173)
M3	Post-doc > senior AP	NO	0	(.)	0	(.)	− 0.048***	(0.0076)
		YES	0.006	(0.0100)	0.018	(0.0111)	− 0.036**	(0.0037)
M4	Junior AP > senior AP	NO	0	(.)	0	(.)	− 0.032**	(0.0097)
		YES	0.028*	(0.0138)	0.049**	(0.0164)	− 0.011	(0.0188)

Robust SE in parentheses: *p < 0.05, **p < 0.01, ***p < 0.001

## Discussion and conclusions

Notwithstanding women’s advance in academia, their loss all along the steps of the career ladder is a well-documented phenomenon. Italy makes no exception with a female presence across the ranks in line with the European average (European Commission, 2024). Up to now, studies based on the Italian Academia have measured the gender promotion gap by focusing on the transitions from associate to full professor and from assistant to associate professor. By means of an original panel dataset, this study is the first to measure the gender penalty in the transition from post-doc to assistant professor thus shedding light on the so-called academic “glass door” (Picardi, 2019).

Results show that women face a small adjusted disadvantage in recruitment which swings around 3–4% according to the model. Gender differences in individual characteristics play a marginal role in explaining the gap, while more interesting results appear when focusing on contextual factors. On this point, the analyses by scientific fields suggest that the gap shows a great variance—swinging from a maximum of − 10% to a non-significant (sometimes positive!) gap across disciplines—which explains the fact that it is rather low in aggregate terms. More precisely, the LS appear the most penalizing macro-field for women, especially due to the bad performance of medicine and biology, while in the PE, the gap is driven by physics and by architecture and construction, but not to—quite surprisingly—math and engineering.

The effects of the feminization of sub-fields on recruitment is not straightforward. The gender gap in recruitment fails short when women are more than 45% only in the transition from junior to senior AP, which is quite coherent with Moss Kanter’s theory. At the same time, the amelioration in the remaining models is rather small and, in any case, does not fail short in gender-balanced sub-fields. More analyses on this point are needed.

Rather than the feminization of decision-making positions, what seems to play a positive lever for women’s recruitment is the fact of working in a department of excellence, especially in the promotion to senior AP. The evidence in this case cautiously goes in the direction of suggesting that by offering better opportunities to all, highly performing and, therefore, “richer” organizations offer better opportunities for women as well.

These analyses have at least two limits. First, as said, the NSQ score is a weak measure of individual productivity, especially for the social and human sciences for which it is not based on bibliometric indicators. Unfortunately, web-scrapping bibliometric information on individual productivity was not possible given the anonymity of the dataset. At the same time, existing web-scrapping methods have proven to be far from being reliable for the SSH because of the limits that available search engines have in terms of coverage of scientific products for this kind of fields (Abramo et al., 2015, 2021; Aksnes & Sivertsen, 2019). Second, the administrative nature of these data did not make it possible to gather all the information wished, i.e., the marital and the parental status, as well as information on parental leaves. This has prevented to conduct more detailed analyses on the gender effects of family characteristics and so to test the hypothesis of the maternity penalty, including its variation across age and number of children, as well as the role of the partner in eventually moderating the relationship between children and promotion for women.

Notwithstanding these limits, the results of these analyses suggest that contextual factors, including field-related and organizational characteristics, play an important role in moderating the gender gap. If this is true, policy interventions should aim at making universities more inclusive towards women rather than empower women to make them “fit” in with the organizations. This could be tackled by implementing positive actions to foster gender awareness, especially among recruiters. Moreover, generous, and horizontal, funds to higher education should be guaranteed. All in all, this paper suggests the important of policies and practices promoting structural change aimed at “adjusting” universities to make them more gender equal.

## Supplementary Information

Below is the link to the electronic supplementary material.

Supplementary file1 (DOCX 366 KB)

## Data Availability

The data that support the findings of this study are available on request from the corresponding author.

## Footnotes

1 The European Commission’s definition of academic grades responds to the need of making the different national career ladders homogeneous across countries, especially with respect to the early phases where national differences are the strongest. And so, the grade C includes both post-docs and assistant professors, the grade B usually refers to associate professors, and the grade A includes full professors (see: European Commission, 2024).

2 Information available here: https://www.mur.gov.it/it/aree-tematiche/universita/le-universita, updated to January 2025 (retrieved April 2nd, 2025). In 2020, according to the dataset used for the present paper, the numbers were slightly different, with 87 public universities, 19 private, and 6 “special university institutes”.

3 Holding the qualification for associate professor is mandatory in order to participate to a public selection for that specific position. Very often, it is a more or less (depending whether it is included in the list of titles to have in the call for application) formal requirement needed to obtain a position of senior assistant professor as well. The reason lie on the fact that—after the end of the contract and an evaluation of the department—the senior assistant professor becomes an associate professor without going through a recruitment process. The promotion is quasi automatic: the real selection in the Italian academic system occurs in the previous phase, that is the transition to the senior AP.

4 More specifically, area 01: mathematics and informatics; area 02: physical sciences; area 03: chemical sciences; area 04: earth sciences; area 05: biological sciences; area 06: medical sciences; area 07: agriculture and veterinary; area 08: architecture and construction; area 09: engineering trades and manufacturing; area 10: archeology, languages and arts; area 11: history, philosophy, psychology, education; area 12: law; area 13: business, administration and statistics; area14: political and social sciences.

5 In bibliometric fields (the STEMM + psychology), the productivity indicators are: (a) the number of articles published in scientific journals; (b) the number of citations; (c) the h-index. In non-bibliometric fields (mainly the SSH), the indicators are: (a) the number of articles published in scientific journals; (b) the number of articles published in "high quality" journals; (c) the number of books. "High-quality" journals are a sub-group of scientific journals. Both of them refer to a periodically updated list of journals defined by the ANVUR based on reputational and impact-related criteria. Each scientific NSQ’s sub-field (“settore concorsuale”) has its own thresholds, that is a minimum-standard value (“valore soglia”), for each indicator. In order to apply to the NSQ, the candidates must have reached at least two out of the three thresholds.

6 Pre-reform AP kept been recruited for a few years after the 2010 reform, with a sharp fall since 2012. At the same time, post-reforms AP appear for the first time in 2011, but for the first couple of years there were few recruitments. In other words, the years 2011–2012 were years of transition were the old contract was progressively replaced by the new one. For this reason, and given the time-frame taken in consideration in this work, the analyses were limited to this last type of contract. 

7 For robustness checks, the same analyses were conducted using logistic models, yielding very similar results (full analyses are available upon request).

8 The variable here is differently coded from the one used in the regressions. In the regression models, the NSQ is a dummy (0 = not holding the qualification; 1 = holding the qualification) where the reference category refers to those who do not hold the qualification, including both those who never applied for the NSQ and those who applied and failed. In these descriptives, these two categories are separated.

9 The citation marks are used in purpose. As mentioned in the literature review, productivity depends on many factors that have to do with the organization of science and its mechanisms of gendered stratification. In other words, if women are systematically disadvantaged in the conditions at the base of scientific productivity, then they will be systematically « less productive» (Gaiaschi, 2022).

10 The correspondence between the two classifications is the following one: the PE include MUR areas n.01 (mathematics and informatics), 02 (physical sciences), 03 (chemical sciences), 04 (earth sciences), 08 (architecture and construction) and 09 (engineering trades and manufacturing); the LS include areas n. 05 (biological sciences), 06 (medical sciences) and 07 (agriculture and veterinary); the SE include areas n. 10 (archeology, languages and arts), 11 (history, philosophy, psychology, education), 12 (law), 13 (business, administration and statistics), and 14 (political and social sciences).
