# Supplementary material for: Breaking the glass door in academia? Looking at the role of scientific fields and contextual factors in moderating the gender gap in recruitment: evidence from Italy
Source: High Educ (Dordr). 2025 Jul 15;91(3):1199–222. doi: 10.1007/s10734-025-01466-4 (PMC13038771; doi:10.1007/s10734-025-01466-4)
Supplement: Supplementary file 1 — Supplementary file1 (DOCX 366 KB) [file 10734_2025_1466_MOESM1_ESM.docx]

**Online Appendix**

**Methodological notes**

**Note on the data collection.** The analyses presented in this work are based on a dataset that draws on administrative and web sources comprising post-docs and assistant professors working in Italy from 2010 to 2020. The administrative data were provided by the MUR (Ministry of University and Research), through the protocol number 0003019.05.05.2022, within the WIRED Project (grant number 898507).

As explained in the main text, they include two different databases: 1) administrative panel micro data on Italian academics; 2) administrative panel micro data on the National Scientific Qualification. Both of them are anonymous. The administrative data collection was extremely long because of several bureaucratic steps, including authorizations and ethical approvals and several “back-and-forth” with the ministerial statistical offices to fine-tune the data. Once obtained, data were codified, harmonized, cleaned and merged with web-scrapped information on organizational performance. The result was a rich and original dataset which has allowed to control for a set of individual and organizational covariates across time. Indeed, three existing, public, data-sources on the Italian academic population (on which many existing studies are based, see: Picardi 2019; De Paola et al. 2018; Marini and Meschitti, 2018; Filandri and Pasqua 2021; Meschitti and Marini 2023) could have been used. However, they do not provide the information I was looking for. Regarding information on the academic population, the open-access website <https://cercauniversita.mur.gov.it/> include micro-data on the academic population but it lacks information on the post-docs across time, which is essential to study the transitions in the early- steps of the career. On the other hand, <https://ustat.mur.gov.it/opendata/> has time-series data, including on post-docs, but on an aggregated level and with very limited information. As for information on the NSQ, public data on candidates, by position and sub-fields, are accessible here: <https://abilitazione.mur.gov.it/public/pubblicarisultati_2023.php>. However, such information, which include the evaluation that the candidates has received, is available to the public only for two months. After that time, it is withdrawn from the website. In summary, the dataset used in this article include information that open-access data can’t provide, that is on post-docs and the NSQ across time.

**Note on time-varying controls.** This dataset includes extensive information tracked across time on individuals (and their socio-demographic and work-related characteristics) and organizations in which individual works. As specified in the paper, controls include age, nationality, the NSQ (y/n), the NSQ score, the scientific field, the geographical location, the university size, the percentage of female full professors (and its squared term), the RQA score, the RQA wave and the 2017 department of excellence.

Time-varying variables include, beside year and age, the qualification (NSQ), the percentage of female full professors by sub-field, the university size, the 2017 excellence ranking, the RQA score. The NSQ variable assigns the value 0 for each year in which the individual does not have the habilitation. If and once he/she obtains it, the variable will show value 1 since the year of obtention onward. In the “2017 excellence ranking” variable, if the department in which the individual works has been granted, it reports value 0 up to 2016 and value 1 from 2017 onward. If the department has never been granted, the variable will show value 0 for each year of observation. In the RQA score, if the University has received an evaluation by the ANVUR concerning the scientific field  in which the individual works, it will report the score obtained in the first wave for the years 2010-2014 (even if in reality the research outputs taken in consideration are related to the years 2011-2014) and the score obtained in the second wave for the years 2015-2010 (even if in reality the correct time frame is 2015-2019). The choice of “stretching” the two evaluation scores in order to include the years 2010 and 2020 respectively has been done in order to reduce missing cases.

The NSQ score has been treated as a time-constant variable. The score is based on the individual’s productivity in the ten years preceding the qualification. For those who have obtained (or attempted) the qualification in the first years of its existence (2012), this may appear as a strong assumption as it means to apply the same productivity score for the years after as well. However, this seemed a reasonable choice in order to reduce the number of missing cases.

**Tables**

**Table A1 – Observations and individuals by gender and position – 2010-2020**

**Table A2 – Transitions from post-docs to AP (individuals) – 2010-2020**

**Table A3 – Men and women by position and year (observations) – 2010-2020**

**Table A4 – Female percentage by scientific field (observations) - 2010-2020**

Note: statistics computed on the full dataset comprising all positions (N: 1,121,360; n: 153,423)

**Table A5 – The gender gap in recruitment: unadjusted and adjusted female coefficients**

**Table A6 - Random intercept multi-level LPM on the position with MUR-codified field**

Legend. Area 01: mathematics and informatics; area 02: physical sciences; area 03: chemical sciences; area 04: earth sciences; area 05: biological sciences; area 06: medical sciences; area 07: agriculture and veterinary; area 08: architecture and construction; area 09: engineering trades and manufacturing; area 10: archeology, languages and arts; area 11: history, philosophy, psychology, education; area 12: law; area 13: business, administration and statistics; area14: political and social sciences.
